# ADAPT: Atlas‐Driven Access to Postmortem Tissue

**DOI:** 10.1002/alz70855_107804

**Published:** 2025-12-24

**Authors:** Jason Webster, Yiqin Alicia Shen, Ali Shojaie, Tung Le, Christine MacDonald, Caitlin S Latimer, C. Dirk Keene, Thomas J Grabowski

**Affiliations:** ^1^ University of Washington, Seattle, WA, USA; ^2^ University of Washington Department of Neurological Surgery, Seattle, WA, USA; ^3^ University of Washington, School of Medicine, Seattle, WA, USA

## Abstract

**Background:**

The validity of research in Alzheimer's Disease and Related Dementias (ADRD) often depends upon the presence or distribution of pathology, assessable through biorepository tissue collected according to a brain sampling protocol (BSP). The digital BSP atlas is recently developed 3D representation of the University of Washington BioRepository and Integrated Neuropathology (BRaIN) protocol for fixed‐tissue sampling on the Montreal Neurological Institute and International Consortium on Brain Mapping 2009b Nonlinear Asymmetric Brain Template (MNI2009b). Here, we link this atlas diverse neuroscience references to locate biorepository tissue samples using arbitrary neuroscience terminology.

**Method:**

Multiple established neuroscience atlases covering anatomy, nuclei, cytoarchitecture, functional connectivity networks, functional regions, and brain areas were registered to MNI2009b. For each atlas, the label, full name, and volume of each label in cubic millimeters within each sample from the BSP were stored in a PyMongo database. A R/Shiny web‐enabled interface was developed to provide intuitive interactive exploration, volume queries, and text‐based search.

**Result:**

ADAPT (**A**tlas‐**D**riven **A**ccess to **P**ostmortem **T**issue) links the digital BSP atlas to a range of digital reference atlases spanning neuroscience attributes. Co‐registration to MNI2009b allows for interactive visualization with freely available neuroimaging software (e.g. FSL, MRICroGL, FreeSurfer). The incorporation labels and full names for diverse atlases enables identifying brain tissue samples containing nearly any neuroscience term.

**Conclusion:**

onnecting the digital BSP atlas to an ecosystem of diverse neuroscience atlases provides a versatile, scalable, user‐oriented, and explicit solution to characterizing and locating brain biorepository resources, optimizing their use for interdisciplinary ADRD research. Incorporating neuroinformatics conventions simplifies incorporating additional atlases and enables compatibility with freely‐available tools for advanced analysis and visualization. The web‐enabled interface facilitates the rapid identification of biorepository samples likely to contain tissue with specific neurosciences attribute along with an estimated volume, lowering the barrier for collaboration between scientists in many fields of AD research. ADAPT can also provide explicit criteria for the optimality of samples for guiding and assessing the precision & accuracy of neuropathological sampling.

